# Electrical Stimulation of the Mesencephalic Locomotor Region Has No Impact on Blood–Brain Barrier Alterations after Cerebral Photothrombosis in Rats

**DOI:** 10.3390/ijms20164036

**Published:** 2019-08-19

**Authors:** Michael K. Schuhmann, Guido Stoll, Lena Papp, Arne Bohr, Jens Volkmann, Felix Fluri

**Affiliations:** Department of Neurology, University Hospital of Würzburg, 97080 Würzburg, Germany

**Keywords:** photothrombotic stroke, deep brain stimulation, mesencephalic locomotor region, blood-brain barrier, tight junctions

## Abstract

Blood–brain barrier (BBB) disruption is a critical event after ischemic stroke, which results in edema formation and hemorrhagic transformation of infarcted tissue. BBB dysfunction following stroke is partly mediated by proinflammatory agents. We recently have shown that high frequency stimulation of the mesencephalic locomotor region (MLR-HFS) exerts an antiapoptotic and anti-inflammatory effect in the border zone of cerebral photothrombotic stroke in rats. Whether MLR-HFS also has an impact on BBB dysfunction in the early stage of stroke is unknown. In this study, rats were subjected to photothrombotic stroke of the sensorimotor cortex and implantation of a stimulating microelectrode into the ipsilesional MLR. Thereafter, either HFS or sham stimulation of the MLR was applied for 24 h. After scarifying the rats, BBB disruption was assessed by determining albumin extravasation and tight junction integrity (claudin 3, claudin 5, and occludin) using Western blot analyses and immunohistochemistry. In addition, by applying zymography, expression of pro-metalloproteinase-9 (pro-MMP-9) was analyzed. No differences were found regarding infarct size and BBB dysfunction between stimulated and unstimulated animals 24 h after induction of stroke. Our results indicate that MLR-HFS neither improves nor worsens the damaged BBB after stroke. Attenuating cytokines/chemokines in the perilesional area, as mediated by MLR-HFS, tend to play a less significant role in preventing the BBB integrity.

## 1. Introduction

After cerebral ischemia, a large number of pathological processes in the microvasculature contribute to the evolution of infarction [1]. A key factor in this context is the breakdown of the blood–brain barrier (BBB) [2], which affects further homoeostasis of ischemic tissue and entails serious clinical events such as hemorrhagic transformation [3]. The BBB consists of brain endothelial cells and pericytes, as well as astrocyte end-feet, and thus, is a highly complex interface between circulating blood and brain parenchyma [4]. Animal models of acute ischemic stroke have shown a timeline for breakdown of the BBB: After interruption of blood supply, an increasing permeability of BBB was observed [2,5]. However, when reperfusion was allowed early, BBB integrity recovered to a certain extent, before a delayed secondary chronic opening of the BBB occurred as a result of an inflammatory response to the ischemic cerebral tissue 24 to 72 h after stroke onset [2]. These findings were corroborated by a recently published study demonstrating that mild disruption of the BBB in stroke survivors is reversible with reperfusion [6]. Thus, the question rises whether targeting BBB dysfunction in the perilesional area of a cerebral ischemia might be a treatment option for ischemic stroke. However, approved pharmacological treatments to prevent BBB after ischemic stroke are not available so far. Interestingly, nonpharmacological approaches, such as electrical stimulation of the sphenopalatine ganglion in a photothrombotic stroke model or deep brain stimulation (DBS) of the anterior thalamic nuclei, reduced BBB leakage [7,8]. These findings might be explained by an attenuated cerebral inflammation or change of the cerebral microvasculature due to electrical modulation of the parasympathetic innervation. Recently, we have shown that DBS of the mesencephalic locomotor region (MLR) improves gait function [9], but also reduces inflammatory processes in the perilesional area of a photothrombotic stroke model [10]. The MLR is a functionally defined region of the brainstem encompassing the cuneiform, pedunculopontine tegmental, and mesencephalic reticular nucleus [11], which is connected indirectly with the cerebral cortex. We hypothesize that high frequency stimulation (HFS) of the MLR might also result in a reduction of BBB disruption due to the attenuated perilesional inflammation, and thus, investigated in the present study whether MLR-HFS: (i) reduces the extravasation of albumin; (ii) decreases the degradation of tight junction molecules, i.e., claudin 3, claudin 5, and occludin; and (iii) reduces the expression of pro-MMP-9.

## 2. Results 

### 2.1. MLR-HFS Does Not Change BBB Leakage and Albumin Extravasation

**Figure 1:**
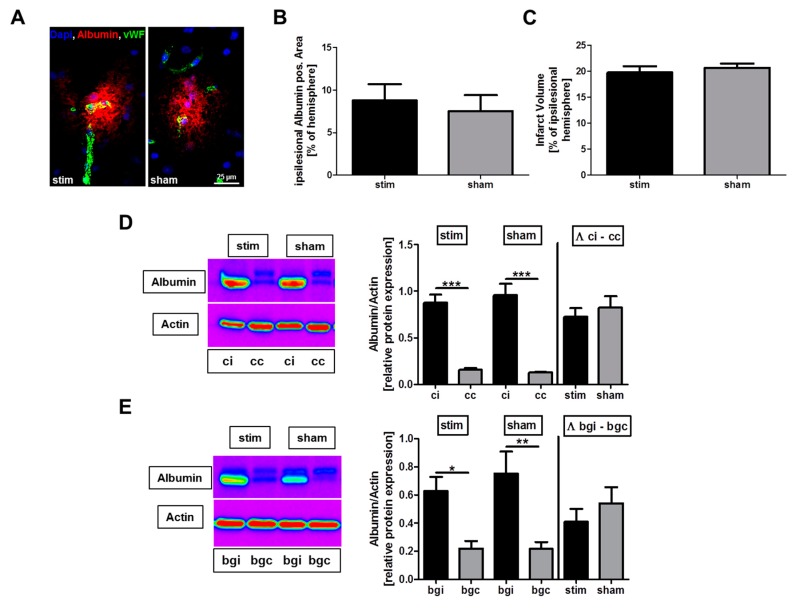
Stimulation of the mesencephalic locomotor region (MLR) in the acute phase after photothrombotic stroke does not alter albumin extravasation and lesion size. (**A**) Representative immunocytologic staining of albumin within the cortical ischemic penumbra of a high-frequency stimulated (stim) and an unstimulated (sham) rat. (**B**) Quantification revealed comparable albumin-positive areas in the ipsilesional hemispheres of unstimulated (sham) and high-frequency stimulated (stim) rats 27 h after photothrombosis (*n* = 6–8/group). Unpaired, two-tailed Student’s *t*-test. (**C**) Infarct volumes are similar (*n* = 6–8/group) between the two treatment groups. Unpaired, two-tailed Student’s *t*-test. (**D**,**E**), Representative anti-albumin Western blot analysis (cc, cortex contralesional; ci, cortex ipsilesional; bgc, basal ganglia contralesional; bgi, basal ganglia ipsilesional) and densitometric quantification of albumin protein expression in the basal ganglial as well as cortical regions 27 h after photothrombosis within the two treatment groups (*n* = 5–7/group). One-way ANOVA, and unpaired, two-tailed Student’s *t*-test. * *p* < 0.05; ** *p* < 0.01; *** *p* < 0.001.

First, infarct volumes were assessed by planimetric analysis of DAPI-stained brain sections of the two groups. Both the sham as well as the stimulated group showed a similar size of infarct volume within the sensorimotor cortex (stim vs. sham: 19.8 ± 1.2% vs. 20.7 ± 2.0%; *p* > 0.05) (Figure 1C).

In the next set of experiments, we investigated immunohistochemically whether MLR-HFS in rats has an impact (i) on BBB leakage in the perilesional area and (ii) on global BBB integrity, i.e., in vessel territories remote from the photochemically induced stroke, as previously reported [5]. Albumin extravasation as a result of BBB leakage was assessed by planimetry in sham-stimulated rats and in rats undergoing MLR-HFS for 24 h, beginning 3 h after induction of photothrombotic stroke. Both the sham and the stimulated group showed a similar albumin-positive area within the ischemic hemispheres (stim vs. sham: 8.8 ± 1.9% vs. 7.5 ± 1.9%; *p* > 0.05), in particular, they did not differ regarding BBB disruption in the area surrounding the photothrombotic lesion (Figure 1A,B).

In a further step, albumin extravasation was examined in the perilesional cortical area as well as in the basal ganglial region using Western blot. This analysis confirmed the aforementioned histological examination: The amount of albumin was significantly increased in the ipsilesional hemisphere, being indicative of post-ischemic BBB disruption. This finding reaffirmed that MLR-HFS did not alter the albumin content neither in the cortical (ipsi: Stim vs. sham: 0.87 ± 0.09 vs. 0.95 ± 0.12; *p* > 0.05) nor in the basal ganglial region (ipsi: Stim vs. sham: 0.63 ± 0.10 vs. 0.75 ± 0.16; *p* > 0.05) (Figure 1A–E).

### 2.2. MLR-HFS Does Not Influence Degradation of Tight Junction Molecules

**Figure 2:**
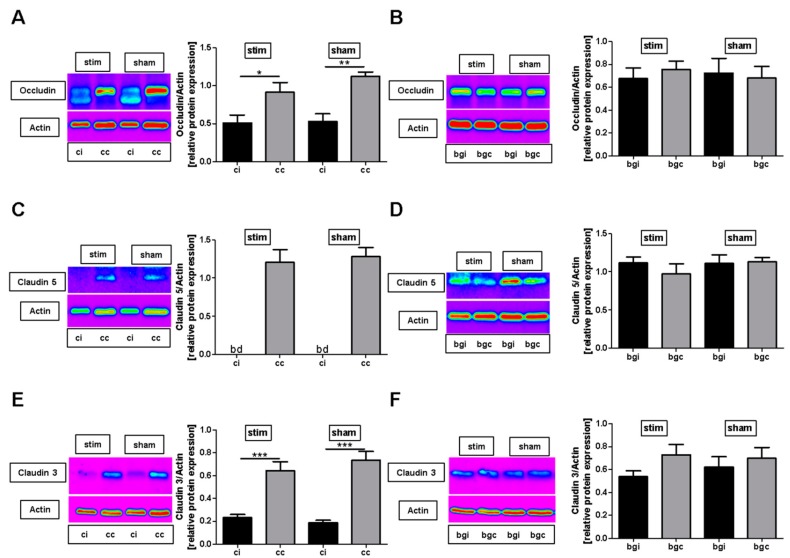
Stimulation of the mesencephalic locomotor region does not modulate photothrombosis-driven tight junction protein degradation. (**A**,**B**), Representative anti-occludin Western blot analysis (cc, cortex contralesional; ci, cortex ipsilesional; bgc, basal ganglia contralesional; bgi, basal ganglia ipsilesional) and densitometric quantification of occludin protein expression in the basal ganglial as well as cortical regions 27 h after photothrombosis of unstimulated (sham) and high-frequency stimulated (stim) rats (*n* = 5–7/group). One-way ANOVA. * *p* < 0.05; ** *p* < 0.01. (**C**,**D**), Representative anti-claudin 5 Western blot analysis (cc, cortex contralesional; ci, cortex ipsilesional; bgc, basal ganglia contralesional; bgi, basal ganglia ipsilesional; bd, beyond detection limit) and densitometric quantification of claudin 5 protein expression in the basal ganglial as well as cortical regions 27 h after photothrombosis within the two treatment groups (*n* = 5–7/group). One-way ANOVA. (**E**,**F**), Representative anti-claudin 3 Western blot analysis (cc, cortex contralesional; ci, cortex ipsilesional; bgc, basal ganglia contralesional; bgi, basal ganglia ipsilesional; bd, beyond detection limit) and densitometric quantification of claudin 3 protein expression in the basal ganglial as well as cortical regions 27 h after photothrombosis within the two treatment groups (*n* = 5–7/group). One-way ANOVA. *** *p* < 0.001.

To assess whether MLR-HFS applied for 24 h protects tight junction molecules from degradation after photothrombotic stroke, occludin, claudin 3, and claudin 5 protein expressions were quantified in cortical and basal ganglial samples using Western blot analysis. In both the stimulated and sham groups, expression of occludin, claudin 3, and claudin 5 was clearly diminished in the perilesional cortex compared to the corresponding region of the contralateral hemisphere. When comparing the perilesional cortex of both groups, no significant difference was detectable regarding the expression of these tight junction proteins (occludin, stim vs. sham: 0.51 ± 0.11 vs. 0.53 ± 0.10, *p* > 0.05; claudin 5, stim vs. sham: Beyond detection level; claudin 3, stim vs. sham: 0.23 ± 0.03 vs. 0.19 ± 0.02, *p* > 0.05) (Figure 2A,C,E). The same was true for expression levels of occludin, claudin 3, and claudin 5 in the basal ganglia regions (occludin, stim vs. sham: 0.68 ± 0.09 vs. 0.72 ± 0.13, *p* > 0.05; claudin 5, stim vs. sham: 1.12 ± 0.07 vs. 1.11 ± 0.11, *p* > 0.05; claudin 3, stim vs. sham: 0.54 ± 0.05 vs. 0.62 ± 0.09, *p* > 0.05) (Figure 2B,D,F).

**Figure 3:**
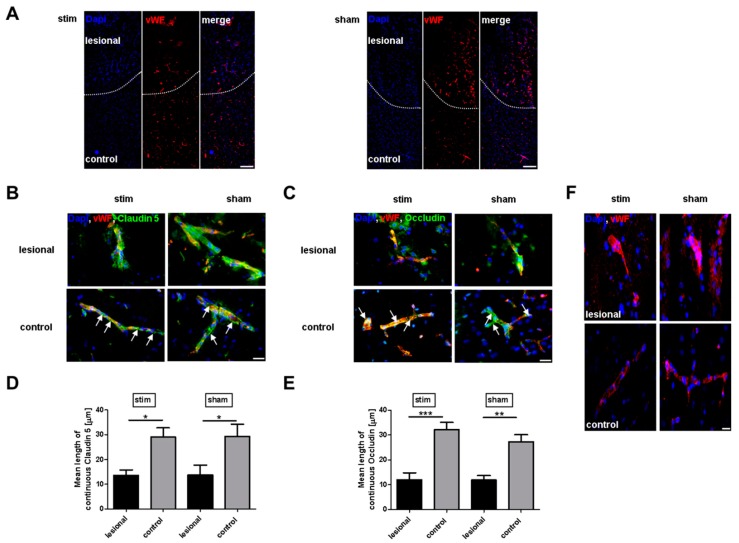
Stimulation of the mesencephalic locomotor region does not modulate photothrombosis-driven tight junction morphology and endothelial activation. (**A**) Representative immunocytologic staining of von Willebrand factor (vWF) in the ischemic hemisphere 27 h after photothrombosis within the two treatment groups. The dashed line indicates the border to the photothrombotic stroke (lesional) (Scale bar: 200 µm). (**B**–**E**) Representative images of anti-claudin 5 and anti-occludin immunohistochemistry in the ipsilatateral (lesional or control) area and quantification of the mean length of the tight junction morphology 27 h after photothrombosis of unstimulated (sham) and high-frequency stimulated (stim) rats (*n* = 5–7/group). One-way ANOVA.* *p* < 0.05; ** *p* < 0.01; *** *p* < 0.001. (Scale bar: 25 µm). (**F**) Representative anti-vWF staining in the ipsilesional hemisphere 27 h after photothrombosis within the two treatment groups (Scale bar: 25 µm).

Next, we applied immunostaining for claudin 5 and occludin to visualize the current state of the vasculature and to provide a detailed insight into the extent of the BBB disruption in the perilesional area. In line with our Western blot analysis, claudin 5 and occludin morphology was clearly changed in the perilesional cortex compared to control, whereas the comparison of the two groups revealed no differences (claudin 5, stim vs. sham: 13.55 ± 2.25 vs. 13.71 ± 4.03, *p* > 0.05; occludin, stim vs. sham: 12.03 ± 2.78 vs. 11.91 ± 1.74, *p* > 0.05), (Figure 3A–E). Importantly, degradation of tight junctions within the ischemic penumbra was accompanied by the activation of endothelial cells, as indicated by a massive change of vWF distribution/expression. Again, no difference became apparent in stimulated and sham-stimulated animals (Figure 3F).

### 2.3. MLR-HFS Does Not Affect Matrix Metalloproteinase-9 Expression after Photothrombosis

**Figure 4:**
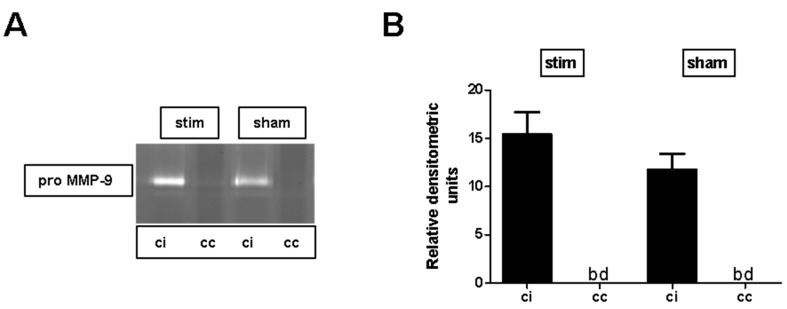
Stimulation of the mesencephalic locomotor region does not modulate photothrombosis-driven pro-matrix metalloproteinase (MMP)-9 expression. (**A**,**B**) Representative experiment of zymography bands (cc, cortex contralesional; ci, cortex ipsilesiona; bd, beyond detection limit) and densitometric quantification of pro-MMP-9 expression in the cortical regions 27 h after photothrombosis of unstimulated (sham) and high-frequency stimulated (stim) rats (*n* = 5–7/group). One-way ANOVA.

To assess whether MLR-HFS applied for 24 h has an impact on metalloproteinases after photothrombotic stroke, pro-MMP-9 protein expression was quantified in cortical samples using zymography. In both stimulated and sham-stimulated animals, expression of pro-MMP-9 was clearly increased in the perilesional cortex compared to the corresponding region of the contralateral hemisphere. When comparing the perilesional cortex of both groups, no difference was found (stim vs. sham: 15.4 ± 2.3 vs. 11.8 ± 1.6, *p* > 0.05) (Figure 4A,B).

## 3. Discussion

This study yielded the following main findings: MLR-HFS, started three hours after induction of a photothrombotic stroke in the sensorimotor cortex of rats and applied continuously for 24 h (i) did not improve nor worsen the hampered BBB due to ischemia; (ii) had no impact on the expression of three main components of the BBB—claudin 3, claudin 5, and occludin; and (iii) had no impact on the expression of pro-MMP-9.

Cerebral ischemia involves different processes that are directed against the BBB integrity, such as activation of endothelial cells, reactive oxygen species, and finally, oxidative stress resulting in a loss of tight junctions [2]. In particular, the importance of inflammatory pathways regarding injury of BBB has been suggested by several studies, whereas chemokines and cytokines are key players in this context [12,13]. Recently, we have shown that MLR-HFS ipsilateral to the lesion enables restoring gait impairment in rats with photochemically induced stroke in the sensorimotor cortex [9], but also attenuates inflammatory processes in the perilesional area when HFS is applied during 24 h in the early course after induction of photothrombotic stroke [10]. Therefore, we hypothesized that this anti-inflammatory effect of MLR-HFS might contribute—at least in part—to the stabilization of the BBB after ischemic stroke. Thus, electrical stimulation would be an alternative to pharmacological therapies targeting BBB stabilization that failed to show any benefit so far. However, the present study demonstrated that MLR-HFS does not attenuate BBB dysfunction in the perilesional area, nor in vessel territories remote from the photothrombotic stroke, as visualized within the basal ganglia. Of note, BBB disruption was not only restricted to the perilesional area surrounding the photothrombotic stroke, but was also apparent in more widespread areas when depicted with a sensitive method such as magnetic resonance imaging with gadoflurine M [5]. In both stimulated and unstimulated animals, a similar change of tight junctions was recognized in the ipsilesional cortex, whereas an alteration of tight junctions was not detectable in the basal ganglia of both groups. On the other hand, we found an increase of albumin in the basal ganglia, independent of electrical stimulation, which might appear contradictory at first sight in regards to the behavior of tight junctions under ischemic conditions. We suggest that BBB disruption remote from the photothrombotic stroke is only attenuated, but is severe enough to open the BBB for proteins of the size of albumin for hours before achieving the initial state again. However, it is much more likely that BBB damage in the perilesional area results in a major extravasation of albumin, which is distributed via the extracellular space over time, and thus, is finally also detectable in the basal ganglia.

In the present study, the effect of MLR-HFS on BBB leakiness after photothromotic stroke was monitored primarily by detecting the loss of tight junction proteins. The role of claudin 3 in contributing to the BBB integrity however is controversial: Whereas some authors have shown an impaired BBB in claudin 3 knockout (KO) mice exhibiting an experimental autoimmune encephalomyelitis [14], Dias and coworkers found that absence of claudin 3 in KO mice did not alter BBB function during neuroinflammation in mice [15]. However, whether the same is true in the paradigm of experimental stroke in rats requires further investigation.

Furthermore, degradation of tight junctions in the area surrounding the photothrombotic stroke was accompanied by the activation of endothelial cells, as indicated by a massive release of vWF. This endothelial vWF exocytosis is probably induced by hypoxia, and results in a rapid recruitment of neutrophils [16,17]. However, this inflammatory phenotype of the BBB obviously cannot be prevented by electrical stimulation, and thus, is not sufficient enough for structural and functional recovery of the BBB.

Whether low frequency stimulation is more likely to result in a stabilization of BBB has not been investigated in this study and has to be addressed in future experiments. Nevertheless, electrical stimulation appears to allow modulating the BBB, depending on the structure that is stimulated— recently, Levi and coworkers have reported that low frequency stimulation of the sphenopalatine ganglion not only diminished infarct volume, but also reduced BBB dysfunction after photothrombotic stroke in rats [7]. Interestingly, when the same ganglion was stimulated at high intensities, an increased BBB permeability was observed in rats [18]. The sphenopalatine ganglion—an important source of parasympathetic innervation to brain vasculature—receives fibers from the brain stem, namely from the reticular formation [19]. Although there are described connections between the MLR and reticular formation [20], and vegetative functions of the MLR are known [21], an innervation of the cerebral vasculature originating from the MLR has not been demonstrated so far. The anterior thalamic nuclei are another cerebral structure that modulates the BBB: A recently published study reported that after electrical stimulation of these nuclei, albumin extravasation was decreased in brains of rats [8].

## 4. Materials and Methods

### 4.1. Animals

Male Wistar rats (Charles River, Sulzfeld, Germany), 10–12-weeks-old, were acclimatized before intervention for 1 week in a room of our animal facility with controlled temperature (22 ± 0.5°C) under a 12 h/12 h light/dark cycle. They were allowed free access to water and food. All animal experiments were approved by the institutional review board of the Julius-Maximilians-University, Würzburg and by the local authorities at the Regierung von Unterfranken, Würzburg, Germany (TVA55.2-2531.01-102/13; 2 October 2013).

### 4.2. Induction of Photothrombotic Stroke

All animals were subjected to photothrombotic stroke as described elsewhere in detail [9]. Under deep anesthesia (isoflurane 2.5% (cp-pharma, Burgdorf, Germany), rats were placed in a stereotactic frame. A template with an aperture covering the right sensorimotor cortex was put on the exposed skull (5 mm anterior to 5 mm posterior and 0.5 mm to 5.5 mm lateral to the bregma). Rose Bengal (Sigma, Darmstadt, Germany) in NaCl 0.9% was administered intravenously and then the sensorimotor cortex was illuminated with a cold light source (Olympus KL1500LCD, Main, Germany) for 15 min.

### 4.3. Microelectrode Implantation

Immediately after induction of photothrombosis, a monopolar electrode was implanted in the ipsilateral MLR (7.8 mm posterior, 2.0 mm lateral and 5.8 mm ventral to the bregma) [9]. The electrode (FHC Inc., Bowddoin, ME, USA) was inserted in the MLR and fixed on the skull with anchor screws and dental cement. A plug (GT-Labortechnik, Arnstein, Germany) was connected with the electrode and attached with additional dental cement. After wound closure, animals were allowed to wake up.

### 4.4. High-Frequency Stimulation of the Mesencephalic Locomotor Region

Rats were divided randomly into a stimulated group (stim-group, *n* = 8) and a nonstimulated group (sham group, *n* = 6). Three hours after photothrombosis onset, MLR-HFS (frequency; 130 Hz; pulse length, 60 µs; monophasic square wave pulses) was begun in the stimulated group and continued for 24 h using a stimulus generator (STG 4002, Multichannel Systems, Reutlingen, Germany). The lowest current evoking locomotion was chosen for the 24 h MLR-HFS [9]. Simultaneously, rats of the sham group were connected with the stimulus generator but no electrical stimulation was applied during 24 h.

### 4.5. Collection of Cerebral Tissue

After injecting pentobarbital into the peritoneum, rats were perfused transcardially with phosphate buffered saline and brains were removed. One millimeter before bregma, a brain section of 2 mm thickness was cut. The cortex comprising the perilesional area, as well as the basal ganglia and the corresponding contralateral regions, were dissected for further analysis.

### 4.6. Immunohistochemistry

Immunohistochemistry of cryo-embedded brain sections was performed using anti-albumin (ab10685, abcam, Cambridge, UK), anti-von Willebrand factor (ab6994, abcam), anti-occludin (611090, BD Transduction Laboratories, San Jose, CA, USA), and anti-claudin 5 (35-2500, Invitrogen, Waltham, MA, USA) antibodies as described recently [22]. Quantification of the albumin-positive area was carried out from 5 subsequent slices per animal with an interval of 100 µm (5-fold magnification). Claudin 5 and occludin staining were quantified using 40-fold magnification by measuring the length of continuous (undisrupted) junctional lines in three optical fields within the regions of interest, applying Image J software version 1.52j (NIH, Bethestda, MD, USA). DAPI staining was used to counterstain cell nuclei and to quantify infarct volumes. Sections were analyzed under a microscope (Leica DMI 8, Leica Microsystems, Wetzlar, Germany) equipped with a charge-coupled device camera.

### 4.7. Western Blot Assays

Western blot analysis was performed according to standard procedures using antibodies against albumin (ab10685, Abcam), occludin (611090, BD Biosciences, San Jose, CA, USA), claudin 3 (34-1700, Thermofisher, Waltgan, MA, USA) claudin 5 (sc-28670, Santa-Cruz, Dallas, TX, USA), and anti-β-actin (A5441, Sigma-Aldrich, Darmstadt, Germany), exactly as described elsewhere [23].

### 4.8. Zymography

The amount of pro MMP-9 in brain lysates was detected by zymography according to standard procedures, exactly as described elsewhere [24].

### 4.9. Statistical Analysis

For statistical analysis, the GraphPad Prism 5.0 software package (GraphPad Software, GraphPad, San Diego, CA, USA) was used. Results are given as mean ± standard error of the mean. Data were tested for Gaussian distribution with the D’Agostino and Pearson omnibus normality test and then analyzed by unpaired, two-tailed Student’s t-test. To test for significant differences between multiple groups, one-way analysis of variance was used, with post hoc Bonferroni adjustment for *p*-values. *p* < 0.05 was considered statistically significant.

## 5. Conclusions

In conclusion, MLR-HFS does not alleviate BBB dysfunction in the paradigm tested here. However, electrical stimulation of distinct brain structures might still be an alternative to modulate BBB function, even under pathological conditions such as ischemic stroke, and might be an additional therapeutic option allowing a more targeted use, as it would be possible with a pharmacological therapy.
